# Brucella Pneumonia Mimicking Pulmonary Tuberculosis: A Case Report

**DOI:** 10.1002/ccr3.70387

**Published:** 2025-04-10

**Authors:** Ammar Chapra, Rashid Kazman, Mhd Baraa Habib, Anas A. Ashour, Mohamed Sadek, Adila Shaukat

**Affiliations:** ^1^ Cardiology Department Hamad Medical Corporation Doha Qatar; ^2^ NYC Health + Hospitals/Woodhull Brooklyn New York USA; ^3^ Internal Medicine Department Hamad Medical Corporation Doha Qatar; ^4^ Infectious Diseases Department Hamad Medical Corporation Doha Qatar

**Keywords:** Brucella, case report, pneumonia, tuberculosis, undulant fever

## Abstract

Brucellosis is a zoonotic infection prevalent in certain regions. It typically presents with nonspecific symptoms but can also cause focal organ involvement, including the musculoskeletal, neurological, or pulmonary systems. Herein, we present a case of a 31‐year‐old male patient who presented with a 5‐day history of fever and cough. Chest X‐ray revealed bilateral patchy consolidations, initially suggesting an infectious etiology such as tuberculosis. Given the patient's background, further investigation confirmed brucellosis‐related pneumonia, an uncommon pulmonary manifestation of brucellosis. Additional workup excluded tuberculosis as a possible cause for the lung findings. He was treated with doxycycline and rifampin, leading to symptom resolution and improvement on follow‐up imaging. This case underscores the importance of considering brucellosis in the differential diagnosis of atypical pneumonia in patients from endemic regions.

## Graphical abstract

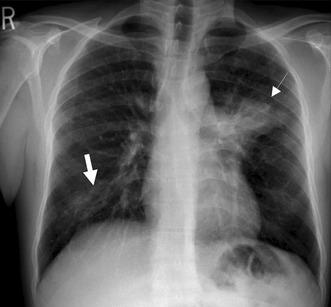

**Summary**

- Brucellosis can affect multiple body systems, including the pulmonary system.
- Although *Brucella* spp. is an uncommon cause of pneumonia, it should be considered a potential etiology in patients with relevant demographic characteristics and symptoms.
- Early recognition and treatment are essential to prevent recurrence and further complications.

## 1 Introduction

Brucellosis is a common zoonotic infection caused by Gram‐negative bacteria known as *Brucella* spp. [1]. Estimates of human brucellosis incidence vary, with recent reports suggesting 1.6–2.1 million new cases annually, significantly higher than the previously cited figure of 500,000 cases per year [2]. It is well known to be present with higher incidence in the Arabian Peninsula, India, Mediterranean region, and Central and South America [2]. The incidence rate among these countries varies, but can reach as high as more than a couple of hundred cases per 100,000 population [3]. With proper antimicrobial treatment, brucellosis has a mortality rate of less than 1%. However, therapeutic failure occurs in up to 15% of cases, highlighting the importance of early diagnosis and treatment [4]. *Brucella* spp. are transmitted to humans from infected animals either by consumption of food products such as unpasteurized dairy products or by contact with their tissue or fluids [5]. The primary source of *Brucella* spp. in the Middle East is considered to be the consumption of raw camel milk [6].

Brucellosis mainly presents with fever that comes in waves (hence it is also known as “undulant fever”), malaise, and arthralgia [1]. Focal involvement is not uncommon and can affect joints and bones (spondylitis, arthritis), cardiovascular system (endocarditis, vasculitis), nervous system (meningoencephalitis), spleen, liver, and kidneys [7]. Pulmonary involvement is rare and is reported in only 2% of brucellosis cases, and brucellosis‐related pneumonia is rather uncommon and is rarely reported in the literature [8]. We are therefore reporting a case of brucellosis‐related pneumonia in an immunocompetent healthy gentleman.

## 2 Case Report

### 2.1 Case History/Examination

A 31‐year‐old South Asian man, with no known chronic medical illnesses, presented to the emergency department complaining of fever and cough that worsened in the 5 days prior to presentation.

His illness began 2 months earlier with a cough productive of whitish sputum associated with an intermittent history of subjective fever. Additionally, he admitted to experiencing severe generalized myalgia and malaise. No medical attention was sought during that time. There was no history of hemoptysis, dyspnea, night sweats, or loss of appetite. However, he reported a history of weight loss, noting that his clothes had become loose. The patient also denied having a skin rash anywhere on his body and did not recall having any joint pain, back pain, abdominal fullness, pain, or swelling in his genitals or perineal area. There was no recollection of contact with anyone with similar symptoms, and he denied having any exposure to a confirmed tuberculosis patient.

The patient worked as a shepherd in the desert tending to mainly camels and cattle. He was a regular consumer of raw camel milk; however, he stopped once his illness began. The patient is a lifetime non‐smoker, and he has no recent history of travel outside the country. His family history was insignificant for any member developing similar symptoms, known to have tuberculosis, or any malignancies. The remainder of his past history was unremarkable.

Upon initial examination, the patient did not appear to be in any respiratory distress. His initial recorded temperature was 39.1°C, pulse was 102/min and regular, respiratory rate was 20/min, blood pressure was 102/60 mmHg, and his pulse oximetry revealed a saturation of 99% while breathing on room air. Systemic examination was remarkable for decreased air entry over the left infra‐scapular area, with no lymphadenopathy or abdominal organomegaly palpable.

### 2.2 Differential Diagnosis, Investigations and Treatment

His initial laboratory investigations revealed a white cell count of 12.3 × 10^9^/L, hemoglobin was 10.9 g/dL, platelet count was 295 × 10^9^/L, sodium level was 135 mmol/L, and potassium level was 3.3 mmol/L. The leukocytosis was associated with an absolute neutrophil count of 9.1 × 10^9^/L and an otherwise normal differential leukocyte count. His C‐reactive protein was 111.6 mg/L, with a Procalcitonin of 0.17 ng/mL, making viral causes more likely as compared to bacterial infections. The remainder of investigations, including a complete metabolic profile and a coagulation profile, were all unremarkable.

**FIGURE 1:**
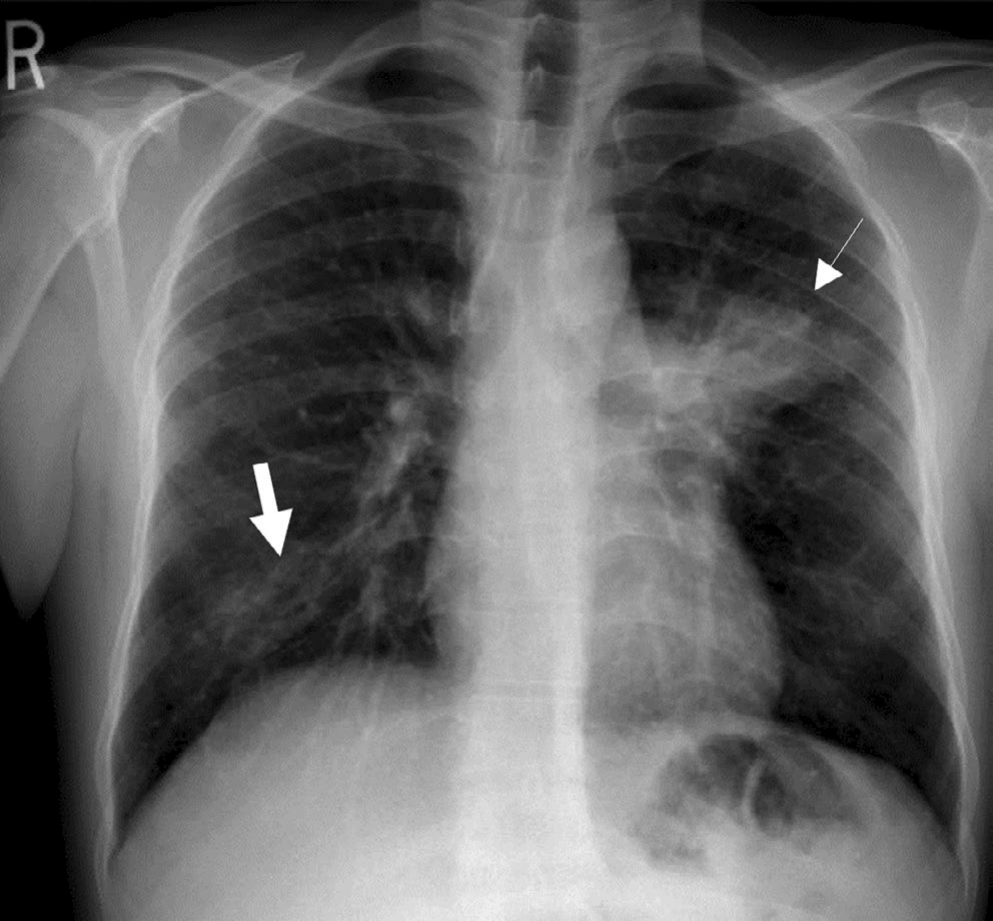
Postero‐anterior radiograph of the chest showing left peri‐hilar patchy consolidation with (thin white arrow) with areas of patchy consolidation at the right lower zone (thick white arrow).

His chest radiograph revealed bilateral patchy consolidation albeit with an unusually located left peri‐hilar consolidation (Figure 1). He was initially started on intravenous (IV) ceftriaxone 2 g once daily for suspected community‐acquired pneumonia after obtaining cultures of his blood, sputum, and urine. He was kept under airborne isolation for strong suspicion of pulmonary tuberculosis given the history and the patient's ethnicity belonging to an endemic area for tuberculosis. Given his occupational history and raw milk ingestion, brucellosis was considered in the differential diagnosis; however, no other organ involvement was evident aside from the chest. Ultrasonography of the abdomen also did not reveal any hepatosplenomegaly or obvious abdominal lymphadenopathy.

During the hospital course, the patient kept spiking high‐grade fever persistently and showed no clinical improvement in symptoms. His initial work‐up of acid‐fast bacilli (AFB) smear and tuberculosis polymerase chain reaction (TB‐PCR) from two sets of sputum samples was negative. The urine culture was unremarkable, and no bacterial growth was reported at 3 days of incubation. Hence, it was decided to widen his anti‐microbial coverage to IV piperacillin‐tazobactam 4.5 g every 8 h; however, the fever did not subside. On the fourth day of his admission, his initial blood culture gram staining was released to be Gram‐negative coccobacilli in aerobic bottle alone, and based on this result, his antibiotics were switched to doxycycline 100 mg, orally, twice daily, along with rifampin 300 mg, orally, twice daily as empiric coverage for brucellosis.

Given the atypical location of the pulmonary infiltrate and the presence of constitutional symptoms, a contrast‐enhanced computed tomography (CT) of the chest was performed to primarily rule out malignancy. The scan demonstrated left upper lobe consolidation and right middle lobe collapse consolidation. Intermittent centrilobular nodules with a tree‐in‐bud pattern were seen more in the left side, suggestive of possible pulmonary tuberculosis among other differentials. There was also significant mediastinal lymphadenopathy present, with few of the small sub‐carinal nodes being calcified. Finally, there were some bronchiectatic airways visible bilaterally.

Subsequently, the patient underwent bronchoscopy during which a bronchial wash and bronchoalveolar lavage (BAL) were performed and revealed predominant neutrophils without any atypical cells. Notably, AFB smear, TB‐PCR, bacterial culture, and fungal culture including *Pneumocystis jirovecii* were all negative, whereas the final *Mycobacterium tuberculosis* culture was pending.

Owing to the close proximity of the mediastinal lymph nodes to the major cardiac vessels and the presence of significant mediastinal lymphadenopathy, it was decided to perform a mediastinoscopy with lymph node biopsy, which the patient underwent with a smooth post‐operative hospital course.

### 2.3 Outcome and Follow‐Up

His final blood culture revealed *Brucella* spp., although further characterization was not possible due to limited resources. The specific serology from peripheral blood displayed a positive Brucella IgG and IgM with a positive antibody titer of 1:320 for both *Brucella abortus* and *Brucella melitensis*. Additionally, the patient became afebrile and reported an improvement in his cough since starting the empiric treatment for brucellosis.

**FIGURE 2:**
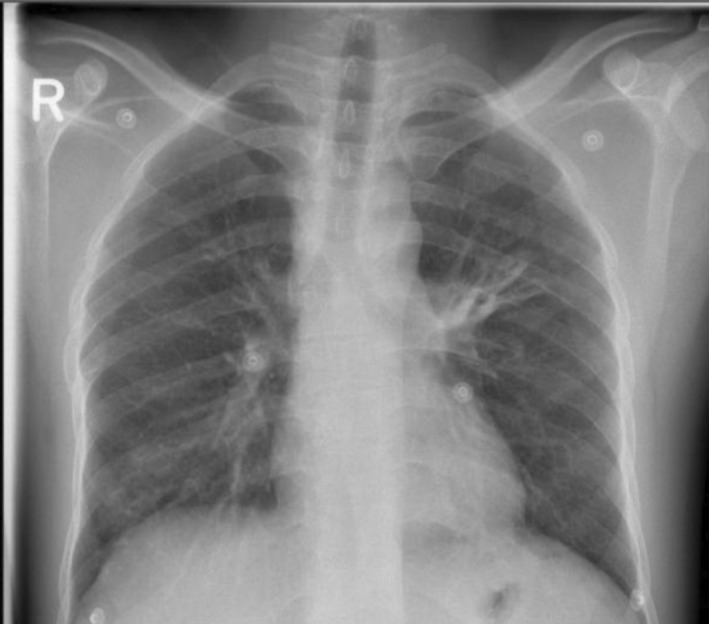
Postero‐anterior radiograph of the chest showing moderate regression in the previously seen left peri‐hilar patchy consolidation.

His follow‐up chest radiograph taken 10 days after the initiation of brucellosis treatment revealed moderate regression in left peri‐hilar consolidation (Figure 2). The patient was discharged with a six‐week course of oral rifampin and doxycycline in good clinical condition, with significant improvement in his cough.

On follow‐up visit, the patient's symptoms had resolved, and his final TB culture came back negative. The results of his lymph node biopsy showed a lymphohistiocytic infiltrate associated with marked anthracosis and no definite morphologic or immunophenotypic features of malignancy involvement.

## 3 Discussion

Brucellosis is a bacterial infection that can involve multiple systems and has a high morbidity for affected patients [2]. Pulmonary involvement is very rare, and inhalation of contaminated aerosols may explain the main mechanism of respiratory system infection [8]. However, some experts also consider spread through the lymphatic system and blood to be a major route for acquiring pulmonary brucellosis [8].

Empyema, lung abscesses, pneumonia, granulomas, solitary nodules, hilar and paratracheal lymphadenopathy are the main pulmonary manifestations that are reported in the literature [8, 9]. Our patient presented solely with brucellosis‐related pneumonia, which is quite uncommon [8].

Different radiological changes have been described in the literature for brucellosis, such as lung abscess, bronchiectasis, pneumonic consolidations, lung nodules, and pulmonary lymphadenopathy [9]. A recent case report described a patient presenting with fever, cough, and hemoptysis [10]. Imaging revealed generalized lymphadenopathy and multiple pulmonary nodules, initially raising suspicion for malignancy. However, further evaluation confirmed a diagnosis of brucellosis [10]. In our patient, the chest radiography revealed left peri‐hilar patchy consolidation with some patchy consolidation in the right lower zone. Keeping in mind the high prevalence of pulmonary TB in the region, the atypical location of the lung consolidation, the constitutional symptoms of the patient, and the rarity of brucellosis causing consolidation, further diagnostic work‐up was sought to exclude other diagnoses. Chest CT scan, bronchoscopy with BAL, and lymph node biopsies were performed to exclude all other possible explanations for his presentation before labeling the patient as brucellosis‐related pneumonia [11, 12].

For individuals suspected of having brucellosis, it is important to obtain blood cultures and serologic tests [7]. A definitive diagnosis is established if the organism is cultured from blood, body fluids (such as pleural fluid, urine, or CSF), or body tissue (like liver biopsy or bone marrow). It can also be confirmed by a fourfold or greater rise in *Brucella* spp. antibody titer between acute and convalescent phase serum specimens obtained two or more weeks apart [7]. In this case, blood culture grew *Brucella* spp., and specific serology from peripheral blood revealed positive Brucella IgM and IgG with a positive antibody titer of 1:320 for both *Brucella abortus* and *Brucella melitensis*.

The main aim for treating brucellosis is to control the illness and prevent complications, relapses, and sequelae [7]. Since *Brucella* spp. are intracellular organisms, successful treatment requires a combination therapy effective in penetrating the cells [7, 13]. Doxycycline with the addition of either rifampin, streptomycin, or gentamicin is the most commonly used regimen for treating brucellosis [7, 13]. Our patient was commenced on doxycycline and rifampin for a period of 6 weeks with an excellent response as evident by resolution of his symptoms in subsequent visits.

This case illustrates the importance of considering brucellosis‐related pneumonia in the differential diagnosis of patients presenting with fever and respiratory symptoms, particularly in endemic regions or in patients with potential exposure risks. Early diagnosis and appropriate treatment are essential to prevent complications and ensure a favorable outcome. Given the rarity of pulmonary involvement, more awareness and research are needed to better understand the full spectrum of brucellosis and its atypical presentations.

## Author Contributions

**Ammar Chapra:** conceptualization, investigation, methodology, writing – original draft, writing – review and editing. **Rashid Kazman:** conceptualization, methodology, writing – original draft, writing – review and editing. **Mhd Baraa Habib:** data curation, investigation, writing – original draft, writing – review and editing. **Anas A. Ashour:** visualization, writing – original draft, writing – review and editing. **Mohamed Sadek:** supervision, writing – review and editing. **Adila Shaukat:** project administration, supervision, writing – review and editing.

## Ethics Statement

Written informed consent was obtained from the patient to publish this report in accordance with the journal's patient consent policy. The case was approved for publication by Hamad Medical Corporation IRB with a protocol number MRC‐04‐21‐1066.

## Consent

Written informed consent was obtained from the patient to publish this report in accordance with the journal's patient consent policy.

## Conflicts of Interest

The authors declare no conflicts of interest.

## Data Availability

Data sharing is not applicable to this article as no datasets were generated or analyzed during the current study.
